# Revascularisation of iatrogenic superior mesenteric artery injury by end to end anastomosis during robot assisted nephrectomy

**DOI:** 10.1016/j.ijscr.2019.09.007

**Published:** 2019-09-19

**Authors:** Sunil Kumar, Shiv C. Navariya, Deepak P. Bhirud, Satish K. Ranjan, Ankur Mittal, Kim J. Mammen

**Affiliations:** Department of Urology, Medical College Building, 6th Floor, All India Institute of Medical Sciences, Rishikesh, Uttarakhand, 249203, India

**Keywords:** Superior mesenteric artery injury, Robot assisted laparoscopic nephrectomy, End to end anastomosis

## Abstract

**Introduction:**

Superior mesenteric artery (SMA) injury is very peculiar to left sided renal surgery. Although it is rare only, most of it is unreported. We report a case of SMA injury during robot assisted laparoscopic nephrectomy, which was managed successfully by end to end anastomosis.

**Case presentation:**

A 19-year-old male patient was undergoing robot assisted laparoscopic simple nephrectomy for pyelonephritic kidney. Because of dense adhesion, SMA was inadvertently clipped and cut. It was recognised intraoperatively and an end to end anastomosis was done by laparotomy.

**Discussion:**

SMA injury is rarely encountered in surgical practice. Most of it occurs following trauma. Iatrogenic SMA injury occurs in case of distorted local anatomy either due to adhesion or bulky tumor in left renal fossa and vicinity.

**Conclusion:**

One should be cautious about proximity of SMA and its possible injury during left nephrectomy and it should be repaired as soon as possible.

## Highlights

• Iatrogenic superior mesenteric artery (SMA) injury is rare and underreported.
• We hereby present a case of SMA injury during robot assisted laparoscopic nephrectomy.
• SMA got clipped and cut due to inability to identify it because of dense perinephric adhesion.
• It was repaired by end to end anastomosis.

## 1 Introduction

A peculiar anatomic relation of left renal hilar vessel is its proximity to superior mesenteric artery (SMA). Due to this reason, SMA is liable to incur injury during left renal surgery, particularly if there is distortion of anatomy due to tumor or adhesion. We report a case, in which SMA was inadvertently cut during robot assisted laparoscopic nephrectomy at a tertiary care academic institute. Fortunately, it was recognised very soon and repaired intraoperatively only. The present work has been reported in line with the SCARE criteria [1].

## 2 Presentation of case

**Fig. 1:**
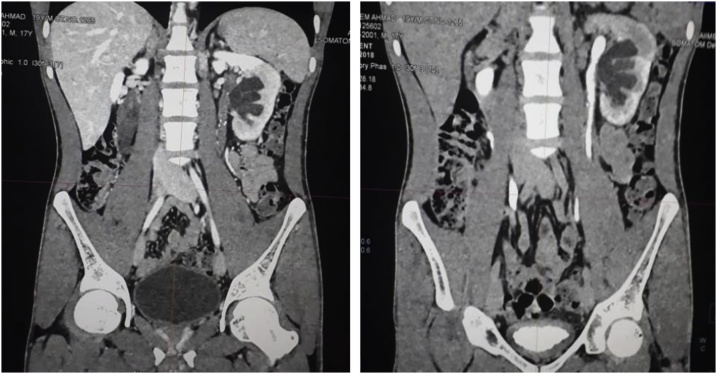
Left, contrast enhanced CT showing hypodense middle and lower pole renal parenchyma. Right, pelvicalyceal system corresponding to middle and lower pole kidney not communicating with ureter.

**Fig. 2:**
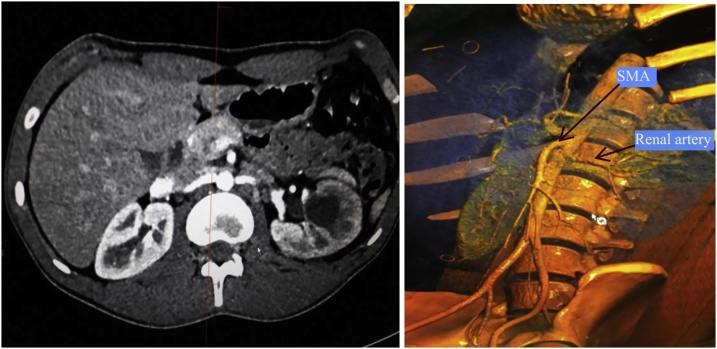
Left, arterial phase of CECT showing renal and superior mesenteric artery, Right, reconstructed angiogram showing close relationship of superior mesenteric artery and left renal artery.

**Fig. 3:**
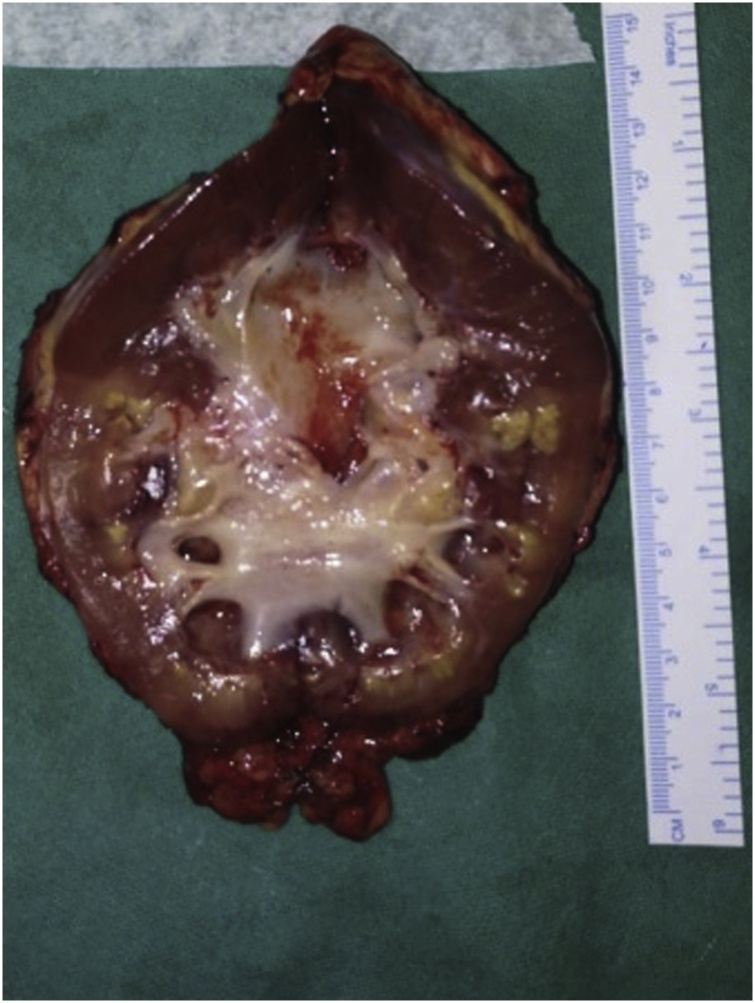
Nephrectomy specimen showing pyelonephritic changes in mid and lower pole.

**Fig. 4:**
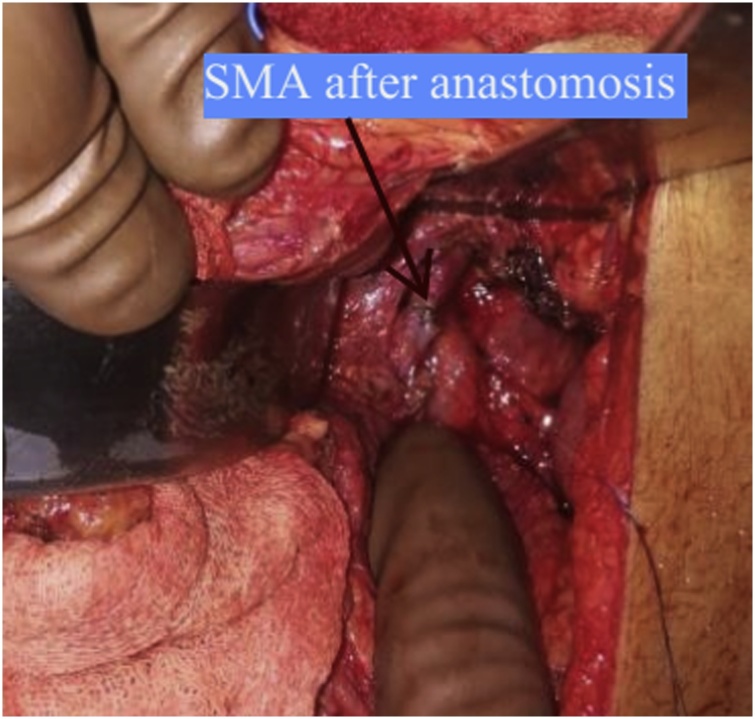
End-to-end anastomosis of superior mesenteric artery.

A 19-year-old male presented with left flank pain and high grade fever. He had undergone open pyelolithotomy 2 months ago at an elsewhere hospital. On evaluation with CT urography, the pelvicalyceal system (PCS) corresponding to mid and lower pole of left kidney was dilated and it was not communicating with pelviureteric junction. The corresponding renal parenchyma of this obstructed renal segment was hypo dense (Fig. 1). Initially, percutaneous drainage of this obstructed segment was done with a pigtail catheter, and the effluent was pus which grew *Klebsiella* on culture. Thereafter, there was no output from PCN. Later on, a nephrostogram combined with retrograde pyeloureterogram showed no communication between mid and lower pole PCS and ureter. Split renal function of left kidney was 21%. As major portion of left kidney was non-functioning and infected, a plan of left nephrectomy was made. Robot assisted laparoscopic nephrectomy was started as usual. Descending colon was reflected medially, gonadal vein and ureter were used as landmark to trace the renal hilum. Because of prior open pyelolithotomy, there was dense perinephric adhesion, and identification of anatomical structures and their alignment was difficult. One artery arising from the aorta was apparently coursing towards the kidney and was assumed to be renal artery. It was clipped and cut. On further dissection, another similar artery was seen. Cross sectional image was reviewed and there was single renal artery. Thus, it was realised that the artery cut earlier was SMA (Fig. 2). Nephrectomy was completed as usual (Fig. 3). Bowel was inspected, but there was no discoloration of bowel. Because of awareness of grave consequences of SMA injury, a decision was made to repair it. Laparotomy was done through midline. Hem o locks on the cut ends of artery were removed after taking control over it by bulldog clamps. To our surprise, there was good projectile back flow from the distal cut end of SMA. An end-to-end anastomosis with 6-0 prolene was done without any need of mobilisation of artery (Fig. 4). Post anastomosis, there was good thrill and pulsation across the anastomosis. Postoperative period was uneventful. At four month follow up, patient is doing well.

## 3 Discussion

Most SMA injuries are encountered following abdominal trauma [2,3]. Iatrogenic SMA injury is rare. It has been reported during pancreatic surgery and nephrectomy for tumors like renal cell carcinoma, transitional cell carcinoma and Wilms tumor [[4], [5], [6], [7]]. SMA supplies entire small bowel and large bowel up to splenic flexure. Communications exist between SMA and inferior mesenteric artery (IMA) through various arterial arcades [[8], [9], [10]]. These collaterals evolve between SMA and IMA in chronically developing stenosis of SMA due to atherosclerotic disease, in which case any injury to either of these major arteries is uneventful. In case of acute injury to SMA, collateral from IMA cannot meet the circulatory requirement of SMA territory. This leads to ischemia of bowel supplied by SMA and this situation is not compatible with life [11]. The change in colour of bowel, palpation of pulse in mesenteric arterial branches and color doppler finding may be misleading. Therefore, an iatrogenic SMA injury should be promptly repaired as was done in our case. SMA injury can be managed by saphenous vein, renal vein patch or by an end to end anastomosis as was done in our case [12,13].

## 4 Conclusion

While doing left nephrectomy in the setting of perinephric adhesion, bulky tumor or lymph nodes with distortion of anatomy, one should be cautious about the possibility of inadvertent SMA injury. Any such injury should always be repaired immediately to avoid its disastrous consequences.

## Sources of funding

No funding from any source.

## Ethical approval

It is exempted as it is a case report and patient’s consent was taken.

## Consent

Patient’s consent was taken.

## Author’s contribution

Study concept or design: Sunil Kumar.

Data collection: Deepak P. Bhirud, Satish k. Ranjan.

Data analysis or interpretation: Sunil Kumar.

Writing the paper: Sunil Kumar, Shiv C. Navariya.

Editing: Kim J. mammen, Ankur Mittal.

## Registration of research studies

Not applicable.

## Guarantor

Sunil Kumar.

## Provenance and peer review

Not commissioned, externally peer-reviewed.

## Declaration of Competing Interest

No conflict of interest.
